# Multimodal integrated approaches in low grade glioma surgery

**DOI:** 10.1038/s41598-021-87924-2

**Published:** 2021-05-11

**Authors:** Tamara Ius, Edoardo Mazzucchi, Barbara Tomasino, Giada Pauletto, Giovanni Sabatino, Giuseppe Maria Della Pepa, Giuseppe La Rocca, Claudio Battistella, Alessandro Olivi, Miran Skrap

**Affiliations:** 1 Neurosurgery Unit, Department of Neurosciences, Santa Maria della Misericordia University Hospital, Piazzale Santa Maria della Misericordia, 15, 33100 Udine, Italy; 2 Institute of Neurosurgery, Fondazione Policlinico Gemelli, Catholic University, Rome, Italy; 3 Department of Neurosurgery, Mater Olbia Hospital, Olbia, Italy; 4 IRCCS “E. Medea,” Polo Regionale del FVG, San Vito al Tagliamento, Pordenone, Italy; 5 Neurology Unit, Department of Neurosciences, Santa Maria della Misericordia University Hospital, Udine, Italy; 6 Department of Medicine, University of Udine, Udine, Italy

**Keywords:** Neuroscience, Oncology, Neurology, CNS cancer

## Abstract

Surgical management of Diffuse Low-Grade Gliomas (DLGGs) has radically changed in the last 20 years. Awake surgery (AS) in combination with Direct Electrical Stimulation (DES) and real-time neuropsychological testing (RTNT) permits continuous intraoperative feedback, thus allowing to increase the extent of resection (EOR). The aim of this study was to evaluate the impact of the technological advancements and integration of multidisciplinary techniques on EOR. Two hundred and eighty-eight patients affected by DLGG were enrolled. Cases were stratified according to the surgical protocol that changed over time: 1. DES; 2. DES plus functional MRI/DTI images fused on a NeuroNavigation system; 3. Protocol 2 plus RTNT. Patients belonging to Protocol 1 had a median EOR of 83% (28–100), while those belonging to Protocol 2 and 3 had a median EOR of 88% (34–100) and 98% (50–100) respectively (*p* = 0.0001). New transient deficits with Protocol 1, 2 and 3 were noted in 38.96%, 34.31% and 31,08% of cases, and permanent deficits in 6.49%, 3.65% and 2.7% respectively. The average follow-up period was 6.8 years. OS was influenced by molecular class (*p* = 0.028), EOR (*p* = 0.018) and preoperative tumor growing pattern (*p* = 0.004). Multimodal surgical approach can provide a safer and wider removal of DLGG with potential subsequent benefits on OS. Further studies are necessary to corroborate our findings.

## Introduction

Adult Diffuse Low-Grade Gliomas (DLGG) are rare genetically heterogeneous tumors accounting for up to 7% of primary brain tumors. DLGG generally affect young adults with no or only slight functional disorders.

Patients with DLGG show an initial subclinical phase, followed by continuous tumor growth, infiltration of eloquent areas, progression to a higher grade of malignancy, which leads to neurological disability and death.

The management of LGGs has radically changed over the time: from a “wait and see” attitude to maximal safe resection as the first step in DLGGs workflow^1–12^. To achieve this purpose, a personalized anatomo-functional planning and intraoperative strategy are crucial to have an optimal balance between a maximal and safe resection.

Because functional neuroimaging tends to show limited reliability, intraoperative direct electrical stimulation (DES), especially in awake patients benefiting from real-time cognitive monitoring, is the best way to increase the extent of resection while sparing eloquent neural networks^13, 14^.

Awake surgery (AS) represents the gold standard for cerebral brain mapping considering that, to date, this is the only technique that permits a direct identification of neural networks^15, 16^. Recent studies have shown that awake mapping in association with real-time neuropsychological testing (RTNT), which reduces the uncertainty of negative mapping, resulted in higher extent of resection (EOR) and preservation of quality of life both for DLGGs involving language and extra-language functional networks^15–17^. Moreover, modern preoperative and intraoperative imaging techniques, along with surgical tools and developments in monitoring techniques, have advanced the potential magnitude of resection in eloquent areas^18^.

These previous studies addressed the impact of each single approach on clinical and EOR parameters^15–17^, but to the best of our knowledge, there are no studies based on the combinatory effect of different techniques.

The principal aim of this investigation was to analyze the effect on the EOR, and consequently on OS, of the evolution of intraoperative techniques, also considering the recent advances of the knowledge of molecular features of DLGG.

## Materials and methods

### Study population

Data from adult patients who underwent surgery for DLGG in a single institution between 2000 and 2018 were retrospectively analyzed.

Patients were enrolled according to the following criteria:

- Age ≥ 18 years
- Preoperative MRI suggestive of supratentorial LGG near or involving the cortical and subcortical eloquent areas
- No previous surgery
- No preoperative chemo- or radiotherapy
- At least 18 months of follow-up
- Objective evaluation of EOR on MRI images in DICOM format based on T2-weighted MRI sequences
- Revision of histopathological specimens according to the 2016 WHO Classification of Tumors of the Central Nervous System
- Intraoperative brain mapping and neurophysiological monitoring.

Needle biopsies were excluded from the study.

The tumor involvement of a cortical or subcortical eloquent was detected according to the relationships of DLGG to eloquent regions of the brain as predicted by the preoperative magnetic resonance imaging scan, functional MRI and Diffusion Tensor Imaging (DTI) data.

The local Ethics Committee, Comitato Etico Unico Regionale del Friuli Venezia Giulia, approved this investigation (protocol N.0036567/P/GEN/EGAS, ID study 2540). Considering that the study was retrospective, written consent to participate in the study was not applicable. Written informed consent was obtained for surgery. Consent in this form was decided and approved by the local ethics committee Regione Friuli Venezia Giulia, Italy. All research was performed in accordance with the Declaration of Helsinki.

### Intraoperative surgical protocol

The surgical procedures were conducted under cortical and subcortical white matter brain mapping, according to the intraoperative technique previously described^19^.

The AS protocol was selected following the standard protocol in use during that specific time period at our institution^17^. In addition to DES, RTNT was applied in the last series during AS^17^.

In this investigation, the data were collected over about 20 years (from 2000 to 2018), during which the intraoperative technical protocol changed.

The following 3 consecutive intraoperative protocols were used during this time period:

- *Protocol 1 (January 2000–December 2004):* patients underwent surgery with the aid of cortico-subcortical DES, neurophysiological monitoring (motor evoked and somatosensorial potential), and intraoperative use of the NeuroNavigation (NN) system.
- *Protocol 2 (January 2005–to present): Protocol 1* plus fMRI/DTI data on the T1-weighted/T2-weighted 3D MRI studies for intraoperative NN.
- *Protocol 3 (January 2011–to present): Protocol 2* plus RTNT in AS.

Patients belonging to *Protocol 1* and *2* were operated on AS or General Anesthesia (GA) based on the specific clinical and neuropsychological status of each subject.

### Volumetric analysis

Magnetic resonance images in DICOM format were used to assess both pre- and postoperative tumor volume by using axial preoperative post-contrast T1-weighted MRI slices, pre and postoperative axial T2-weighted MRI studies. All pre- and postoperative tumor segmentations were performed manually across all MRI slices using the OsiriX software tool^20^.

The preoperative tumor growing pattern (infiltrative vs. expansive) was expressed as ΔT2T1 index that is preoperative volumetric tumor volume on T2-weighted MRI images—preoperative volumetric tumor volume on T1-weighted images^6, 20^. As previously described, higher levels of preoperative ∆T2T1 MRI index represent the prevalence of the diffusive growing mechanism with the tendency to infiltrate the functional cortical areas and subcortical pathways, thus limiting the achievable resection.

The postoperative EOR was evaluated by using 3D T2-weighted MRI axial images as follows: (pre-operative tumor volume – post-operative tumor volume)/(pre-operative tumor volume)^20^.

### Histological and molecular analysis

All histological samples were reviewed according to the 2016 World Health Organization (WHO) classification^21^. Molecular markers were evaluated as previously described^22^.

### Statistical analysis

Statistically significant differences on distribution were evaluated performing chi-squared test for categorical variables, and t-test, Wilcoxon rank-sum tests or Kruskal–Wallis for continuous variables, as appropriate.

Overall Survival (OS), progression free survival (PFS) and malignant progression free survival (MPFS) were estimated using the Kaplan–Meier approach. Univariate and multivariate Cox regression models were performed to identify the association between any variable and OS, PFS and MPFS as outcome variables, after the proportional hazards assumption had been verified.

The primary endpoints were EOR and OS differences among the three groups.

Retention in the stepwise model required the variable to be significant to a level of *p* = 0.05 in a multivariate analysis^23^.

The Spearman’s rank correlation coefficient was computed to define the relationship between pre-operative ΔT2T1 MRI index and postoperative residual tumor computed on T2-weighted MR images.

All analyses were conducted using Stata/SE software (version 14.0 Stata Corp.), and data were presented as HRs and 95% CIs.

### Ethical approval

The local Ethics Committee, Comitato Etico Unico Regionale del Friuli Venezia Giulia, approved this investigation (protocol N.0036567/P/GEN/EGAS, ID study 2540).

### Informed consent

Considering that the study was retrospective, written consent to participate in the study was not applicable. Written informed consent was obtained for surgery.

## Results

### Study population

Three hundred and fifty-two adult patients underwent surgery at our institution for primary supra-tentorial LGGs. A total of 288 patients met the inclusion criteria. Patient demographic, clinical and radiological data are summarized in Table 1. According to clinical and neuropsychological status of each subject, AS was selected in 175 cases, GA was used in 113 cases.

**Table 1:** Clinical, radiological and molecular characteristics of the study population.

Parameter	Value
No. of patients	288
Age	37.5 years (18–74)
**Sex**	
Male	168 (58.33)
Female	120 (41.67)
**Clinical onset**	
Generalized seizure	160 (55.56)
Focal seizure	80 (27.78)
Incidental	36 (12.5)
Others (neurological deficits, mood changes)	12 (4.17)
**Tumor side**	
Left	160 (55.56)
Right	128 (44.44)
**Tumor site**	
Frontal (inferior frontal gyrus, premotor cortex, middle frontal gyrus, Broca’s area)	118 (40.97)
Parietal	34 (11.81)
Temporal	54 (18.75)
Insular	77 (26.74)
Occipital	5 (1.74)
Preoperative tumoral volume on T2WI (cm^3^)	40 (22–68)
**Preoperative ΔT2T1 MRI index, cm**^**3**^	
ΔT2T1 MRI index	10 (0–20)
**≥ **15	116 (40.38)
**< **15	172 (59.72)
**Anesthesia**	
Awake	175 (60.77)
General	113 (39.23)
**Intraoperative surgical protocol**	
Protocol 1	77 (26.74)
Protocol 2	137 (47.57)
Protocol 3	74 (25.69)
**EOR %**	
≥ 90	146 (50.69)
70–89	101 (35.07)
< 70	41 (14.24)
Median EOR %	90 (28–100)
Protocol 1: Median EOR %	83 (28–100)
Protocol 2: Median EOR %	88 (34–100)
Protocol 3: Median EOR %	100 (50–100)
Awake surgery	90 (28–100)
General anesthesia	85 (34–100)
**Median EOR % in awake surgery subgroup**	
Protocol 1, only awake surgery: Median EOR %	85 (28–100)
Protocol 2, only awake surgery: Median EOR %	90 (49–100)
Protocol 3, only awake surgery: Median EOR %	100 (50–100)
**Median EOR % stratified by molecular class**	
DA IDH 1/2wt	83 (34–100)
DA IDH 1/2mt	87 (28–100)
OD IDH 1/2mt,1p19q cod	92 (55–100)
**Post-operative residual tumor volume on T2WI (cm**^**3**^**)**	
< 10	189 (65.63)
10–19	51 (17.71)
20–29	23 (7.99)
≥ 30	25 (8.68)
Median	5 (0–125)
**Diagnosis WHO 2016**	
DA IDH-wt	34 (11.8)
DA IDH-mt	163 (56.6)
OD IDH-mt 1p19q cod	91 (31.6)
**Clinical follow up**	
Median follow up (months)	71 (18–239)
Patient deaths	141 (48.96)
n° w/disease progression	199 (69.1)
Median time to progression (months)	46 (4–194)
n° w/malignant progression	159 (55.21)
Median time to malignant progression (months)	60 (6–239)

Characteristics of the study population are described using means (standard deviation) or median and range for continuous variables, number of cases with relative percentages reported in parentheses for categorical variables. *ΔT2T1 MRI index* volumetric difference between preoperative tumor volumes on T2 and T1 weighted MRI images, *EOR* extent of surgical resection, *DA IDHwt* Diffuse Astrocytoma Isocitrate Dehydrogenase wild type, *DA IDHmt* Diffuse Astrocytoma Isocitrate Dehydrogenase mutated, *OD IDHmt 1p19q cod* Oligodendroglioma Isocitrate Dehydrogenase mutated, 1p 19q codeleted.

Patients were surgically treated as follows:

- *Protocol 1*: 77 patients (26.74%).
- *Protocol 2*: 137 patients (47.57%).
- *Protocol 3*: 74 patients (25.69%).

Overall, new postoperative deficits were noted in a total of 100 patients (34.72%). The incidence of permanent postoperative deficits was relatively low in all protocol groups. Post-surgical deficits were stratified according to each surgical protocol (Table 2).

**Table 2:** Neurological impairment in the immediate post-operative period and 6 months after surgery in the general population and according to intraoperative protocol.

	General population	Protocol 1	Protocol 2	Protocol 3
**Total population**	288	77	137	74
**PO deficit—total**	100 (34.72%)	30 (38.96%)	47 (34.31%)	23 (31.08%)
Aphasia and hemiplegia	3 (1.04%)	0 (0%)	1 (0.73%)	2 (2.7%)
Dysarthria	14 (4.86%)	2 (2.6%)	6 (4.38%)	6 (8.11%)
Dysphasia	12 (4.17%)	3 (3.9%)	5 (3.65%)	4 (5.41%)
Dysphasia and hemiplegia	1 (0.35%)	0 (0%)	1 (0.73%)	0 (0%)
Dysphasia and hemiparesis	1 (0.35%)	0 (0%)	0 (0%)	1 (1.35%)
Dysphasia and upper limb paresis	2 (0.69%)	0 (0%)	0 (0%)	2 (2.7%)
Dysarthria and upper limb paresis	4 (1.39%)	2 (2.6%)	1 (0.73%)	1 (1.35%)
Dysphasia and upper limb paresthesia	1 (0.35%)	0 (0%)	1 (0.73%)	0 (0%)
Hemiplegia	9 (3.13%)	4 (5.19%)	5 (3.65%)	0 (0%)
Hemiparesis	11 (3.82%)	5 (6.49%)	4 (2.92%)	2 (2.7%)
Lower limb paresis	11 (3.82%)	5 (6.49%)	6 (4.38%)	0 (0%)
Upper limb paresis	12 (4.17%)	2 (2.6%)	7 (5.11%)	3 (4.05%)
Upper limb plegia	1 (0.35%)	0 (0%)	1 (0.73%)	0 (0%)
Hypoesthesia of the Hemisoma	1 (0.35%)	1 (1.3%)	0 (0%)	0 (0%)
Ataxia	1 (0.35%)	1 (1.3%)	0 (0%)	0 (0%)
Lower limb paresthesia	3 (1.04%)	0 (0%)	3 (2.19%)	0 (0%)
Lower limb paresthesia and upper limb paresis	1 (0.35%)	0 (0%)	1 (0.73%)	0 (0%)
Parestesia of the hemisoma	4 (1.39%)	0 (0%)	3 (2.19%)	1 (1.35%)
SMA syndrome	8 (2.78%)	5 (6.49%)	2 (1.46%)	1 (1.35%)
**6-m deficit—total**	12 (4.17%)	5 (6.49%)	5 (3.65%)	2 (2.7%)
Aphasia and hemiplegia	2 (0.69%)	0 (0%)	1 (0.73%)	1 (1.35%)
Dysarthria	1 (0.35%)	1 (1.3%)	0 (0%)	0 (0%)
Dysphasia	2 (0.69%)	0 (0%)	1 (0.73%)	1 (1.35%)
Hemiplegia	6 (2.08%)	3 (3.9%)	3 (2.19%)	0 (0%)
Hemiparesis	1 (0.35%)	1 (1.3%)	0 (0%)	0 (0%)

Table showing the presence of neurological impairment in the immediate post-operative period and 6 months after Diffuse Low-Grade Glioma surgery in the general population and for each intraoperative Protocol.

Protocol 1: Brain mapping and Direct Electrical Stimulation.

Protocol 2: Protocol 1 plus overlap of functional MRI/DTI imaging on a NeuroNavigation system.

Protocol 3: Protocol 2 plus Real-Time Neuropsychological Testing.

*PO deficit* neurological deficit in the immediate Post-Operative period, *6-m deficit* neurological deficit 6 months after surgery.

In detail, new deficits with *Protocol 1, 2* and *3* were noted respectively in 38.96%, 34.31% and 31.08% of cases in the immediate post-operative period and in 6.49%, 3.65% and 2.7% of patients 6 months after surgery.

No intra or postoperative mortality was observed.

### EOR and surgical protocol

**Figure 1:**
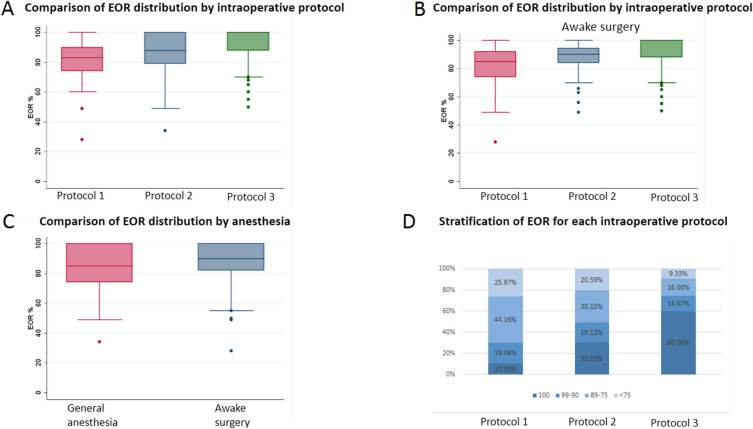
Difference achieved in tumor resection according to the intraoperative protocol used. (**A**) shows the median EOR achieved in patients operated with general anesthesia and awake craniotomy. In (**B**) the EOR data are stratified according to the intraoperative surgical protocol. Protocol 1 (Mapping) had a median EOR of 83%, while Protocol 2 (Mapping + DTI + fMRI) had a median EOR of 88% and Protocol 3 had a median EOR of 100%. (**C**) displays the median EOR achieved in the subgroup of patients who underwent awake craniotomy: Protocol 1 (Mapping) 85%, Protocol 2 (Mapping + DTI + fMRI) 90% and Protocol 3 100%. The *circles* represent the outlier values. (**D**) is a bar chart representing the distribution of EOR as a categorical variable in the three surgical protocols. *EOR* Extent Of Resection.

The median EOR was 90% (28–100) in the study population. The mean EOR resulted to be statistically different between patient operated with AS when compared to those operated under GA (*p* = 0.006). Among patients operated on AS, mean EOR was higher in those in which the *Protocol 3* was applied (*p* = 0.001) (Fig. 1). Figure 1D stratifies the EOR subgroups according to the intraoperative protocol.

A significant association was found between postoperative tumor volume on T2-weighted MRI images and ΔT2T1 value (rho = 0.788, *p* < 0.001).

### OS, PFS and MPFS

Data on 3-,5-, and 10 years estimated OS, PFS and MPFS are summarized in Table 3.

**Table 3:** Overall survival, progression free survival and malignant progression free survival at 3, 5 and 10 years after surgery.

	Overall survival % (95%CI)	Progression free survival % (95%CI)	Malignant progression free survival % (95%CI)
3 years	87.58 (83.04–90.96)	69.23 (63.39–74.33)	80.17 (74.95–84.41)
5 years	75.80 (70.01–80.62)	47.77 (41.41–53.85)	64.88 (58.64–70.43)
10 years	43.69 (36.35–50.79)	15.39 (10.37–21.33)	32.85 (26.04–39.80)

Table showing overall survival, progression free survival and malignant progression free survival at 3, 5 and 10 years after surgery.

**Figure 2:**
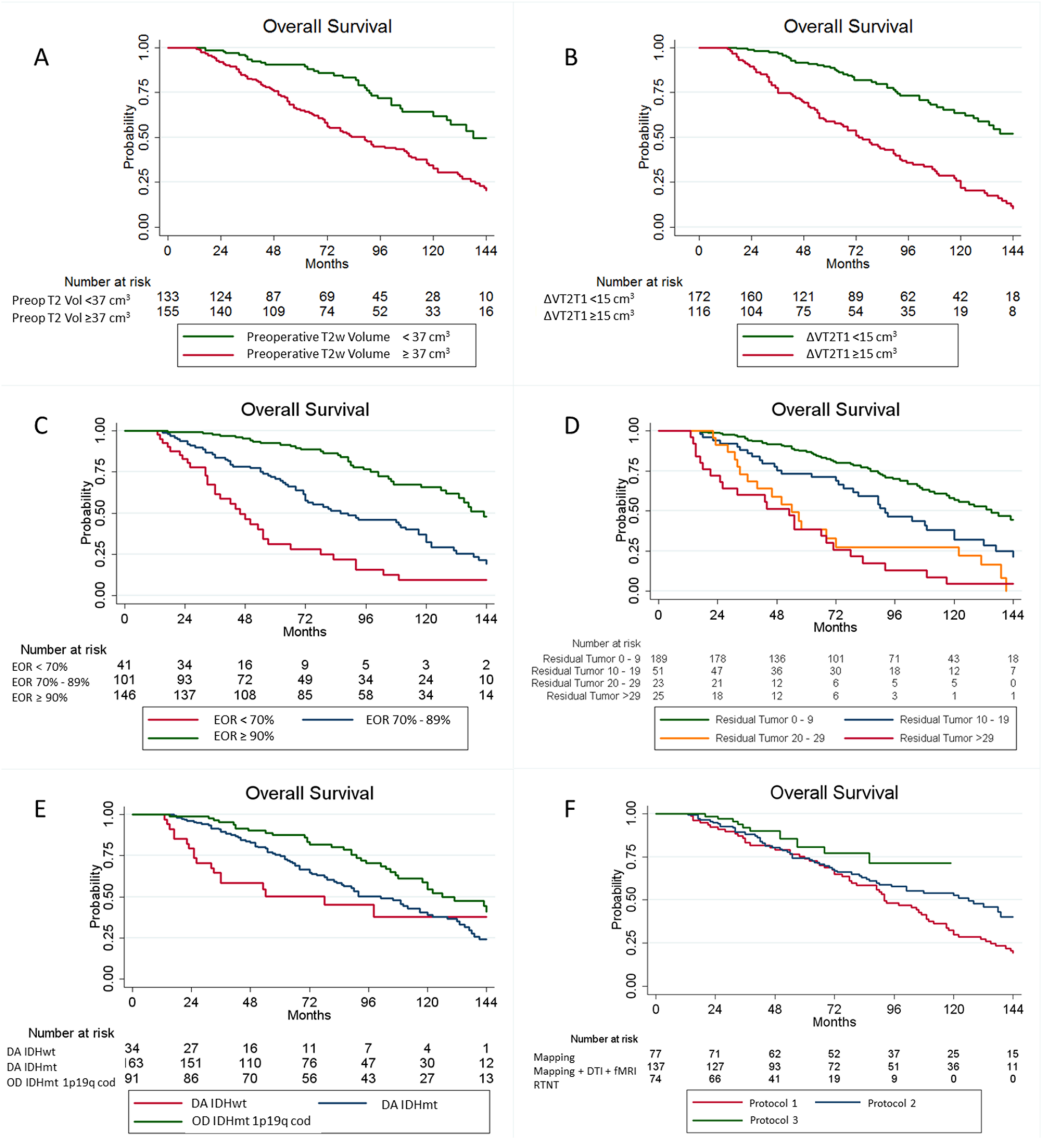
Kaplan–Meier curves displaying overall survival (OS) according to the preoperative tumor volume (**A**), preoperative tumor growing pattern, as expressed by ΔT2T1 (**B**), extent of resection (**C**), intraoperative protocol (**D**), residual tumor (**E**) and molecular class (**F**). *Preop T2 Vol*  preoperative tumor volume computed on T2-weighted images, *ΔT2T1* preoperative tumor volume segmented on T2-weighted MRI images – preoperative tumor volume segmented on T1-weighted images, *EOR* extent of resectionm *DA IDHwt* Diffuse Astrocytoma Isocitrate Dehydrogenase wild type, *DA IDHmt* Diffuse Astrocytoma Isocitrate Dehydrogenase mutated, *OD IDHmt 1p19q cod* Oligodendroglioma Isocitrate Dehydrogenase mutated, 1p 19q codeleted.

Specifically, results of multivariate analysis showed that OS is independently associated with preoperative tumor growing pattern expressed by ΔT2T1 (*p* = 0.004), molecular class (*p* = 0.028), EOR (*p* = 0.018) (Table 4, Fig. 2). The intraoperative protocol was not significantly associated with OS after multivariate analysis probably as a consequence of the more overwhelming effect of EOR.

**Table 4:** Predictors of overall survival univariate and multivariate analyses.

Variable	Univariate analysis			Multivariate analysis		
	Odds ratio	95% CI	*p* value	Odds ratio	95% CI	*p* value
Age (years)	1.017	1.003–1.031	**0.014**	1.010	0.993–1.041	0.248
**Anesthesia**						
General	1					
Awake	0.801	0.571–1.124	0.199			
**Intraoperative protocol**						
1	1					
2	0.660	0.465–0.937	**0.020**	0.952	0.616–1.472	0.826
3	0.448	0.238–0.842	**0.013**	1.174	0.561–2.455	0.671
**PO deficit**						
No deficit	1					
Presence of neurological deficit	1.858	1.322–2.610	**< 0.001**	1.406	0.912–2.168	0.123
**6-m deficit**						
No deficit	1					
Presence of neurological deficit	1.898	1.114–3.235	**0.018**	0.797	0.397–1.600	0.523
**Clinical onset**						
Generalized seizure	1					
Focal seizure	1.395	0.978–1.991	0.067	1.509	0.999–2.280	0.051
Neurological impairment	3.300	1.036–10.512	**0.043**	1.827	0.397–8.422	0.439
Incidental	0.121	0.030–0.491	**0.003**	0.518	0.117–2.288	0.386
Headache	1.977	0.621–6.293	0.249	1.589	0.443–5.701	0.478
**Side**						
Left	1			1		
Right	0.747	0.535–1.043	0.087	0.944	0.636–1.401	0.774
**Tumor site**						
Insula	1			1		
Frontal lobe	0.848	0.571–1.260	0.415	1.087	0.643–1.837	0.756
Parietal lobe	1.267	0.756–2.122	0.369	1.173	0.618–2.229	0.625
Temporal lobe	0.611	0.352–1.058	0.079	0.907	0.476–1.728	0.766
**Histological diagnosis (WHO 2016)**						
OD IDHmt 1p19q cod	1			1		
DA IDHwt	2.830	1.616–4.959	**< 0.001**	2.663	1.271–5.583	**0.009**
DA IDHmt	1.700	1.149–2.516	**0.008**	1.680	1.059–2.666	**0.028**
**Radiological features**						
Preoperative tumoral volume computed on T2-weighted images, cm^3^	1.008	1.005–1.011	**< 0.001**	0.998	0.991–1.005	0.615
Preoperative MRI Index ΔVT2-T1	1.039	1.030–1.048	**< 0.001**	1.026	1.008–1.044	**0.004**
Residual tumor, cm^3^	1.017	1.012–1.023	**< 0.001**	0.996	0.978–1.015	0.662
EOR (continuous variable)	0.956	0.946–0.965	**< 0.001**	0.975	0.956–0.996	**0.018**
**Biological features**						
p53	1.314	0.909–1.901	0.147			
ATRX	0.880	0.604–1.282	0.506			

Table showing the influence of different factors on the OS rates as per univariate survival analysis and multivariate analysis on the entire patient’s cohort. (*p* value < 0.05 at Log-rank test). Boldfacing represent statistically significant results (*p* < 0.05). *CI* confidence interval, *p value* level of marginal significance, *PO deficit* post-operative deficit, *6-m deficit* deficit 6 months after surgery, *MRI* magnetic resonance image, *IndexΔVT1/T2* ratio between pre-operative tumoral volume on postcontrast T1-weighted and T2-weighted images, *EOR* extent of resection, *RT* radiotherapy, *CT* chemotherapy, *MGMT* O6-alkylguanine DNA alkyltransferase, *OS* overall survival.

Figure 2 shows the OS Kaplan–Meier curves stratified according to preoperative tumor volume, ΔT2T1, EOR, volume of residual tumor, molecular class and intraoperative protocol.

Data on 3-, 5-, and 10 years estimated OS, PFS and MPFS were also stratified according to the EOR achieved and molecular class (Tables 5, 6).

**Table 5:** Overall survival, progression free survival and malignant progression free survival at 3, 5 and 10 years after surgery according to extent of resection.

	EOR 100%	EOR 99–85%	EOR < 85%
**Overall survival**			
3 years	97.72 (91.18–99.43)	95.46 (88.36–98.27)	71.61 (61.58–79.46)
5 years	94.92 (86.92–98.08)	84.79 (75.24–90.88)	52.46 (41.83–62.03)
10 years	86.58 (71.80–93.92)	45.28 (33.52–56.31)	19.21 (11.28–28.74)
**Progression free survival**			
3 years	90.83 (82.49–95.31)	69.93 (59.27–78.30)	49.26 (38.99–58.73)
5 years	70.94 (58.65–80.18)	49.02 (38.20–58.97)	26.82 (18.09–36.33)
10 years	46.49 (31.41–60.25)	11.28 (5.47–19.44)	3.76 (0.77–10.95)
**Malignant progression free survival**			
3 years	94.26 (86.75–97.57)	87.77 (79.00–93.04)	60.57 (50.18–69.45)
5 years	91.12 (82.08–95.71)	67.93 (57.01–76.64)	40.92 (30.79–50.77)
10 years	72.74 (54.86–84.47)	26.87 (17.31–37.36)	14.69 (7.76–23.71)

Table showing overall survival, progression free survival and malignant progression free survival at 3, 5 and 10 years after surgery according to extent of resection. Results are displayed as percentage (95% confidence interval).

EOR = extent of resection.

**Table 6:** Overall survival, progression free survival and malignant progression free survival at 3, 5 and 10 years after surgery in patients with total resection, stratified according to molecular diagnosis (WHO 2016).

	Among patients with EOR = 100% % (95% CI)		
	DA IDHwt	DA IDHmt	OA IDHmt 1p19q cod
Number of patients	12	45	37
**Overall survival %(95%CI)**			
3 years	91.67 (53.90–98.78)	97.56 (83.92–99.65)	100.00 (-)
5 years	91.67 (53.90–98.78)	91.76 (76.39–97.29)	100.00 (-)
10 years	91.67 (53.90–98.78)	87.59 (69.47–95.29)	86.54 (55.83–96.48)
**Progression free survival %(95%CI)**			
3 years	91.67 (53.90–98.78)	88.15 (73.83–94.89)	93.94 (77.69–98.47)
5 years	82.50 (46.09–95.33)	62.40 (43.68–76.45)	77.18 (55.45–89.25)
10 years	68.75 (29.07–89.26)	51.51 (30.79–68.82)	26.23 (7.52–50.06)
**Malignant progression free survival %(95%CI)**			
3 years	91.67 (53.90–98.78)	92.85 (79.43–97.64)	96.67 (78.61–99.52)
5 years	91.67 (53.90–98.78)	85.97 (68.99–94.03)	96.67 (78.61–99.52)
10 years	91.67 (53.90–98.78)	71.05 (42.29–87.30)	70.86 (41.90–87.25)

Table showing overall survival, progression free survival and malignant progression free survival at 3, 5 and 10 years after surgery according to molecular diagnosis (WHO 2016), in patients with Extent of Resection = 100%.

*EOR* extent of resection, *DA IDHwt* Diffuse Astrocytoma Isocitrate Dehydrogenase wild type, *DA IDHmt* Diffuse Astrocytoma Isocitrate Dehydrogenase mutated, *OD IDHmt 1p19q cod* Oligodendroglioma Isocitrate Dehydrogenase mutated, 1p 19q codeleted.

## Discussion

In this retrospective study, which included 288 adult patients surgically treated for DLGG, the use of different intraoperative protocols implemented in a large single center series over time was assessed based on neurological outcomes, EOR and OS.

The most relevant results we focused on were:

1. The evolution of the intraoperative surgical protocol over time led to an improvement in EOR.
2. The highest percentage of total resection (EOR 100%) was obtained amongst patients treated with *Protocol 3* (DTI and fMRI data loaded in NN + AS + RTNT).
3. Preoperative ΔT2T1 MRI index, EOR and the molecular class are confirmed as independent predictors for OS.
4. Tumor recurrence was also seen in patients that underwent radical resection and amongst all molecular classes.

### The role of EOR

In the last decades, DLGG management paradigms have evolved from “wait and see” strategy to a more active interventional approach that aims at reducing the risk of malignant transformation. Numerous studies strongly support that a more extensive resection of DLGGs is associated with improved overall survival time and tumor progression free survival time^1, 3, 6, 11, 12, 24–38^.

The milestone of LGGs surgery is the concept of maximal safe resection^5, 17, 39, 40^.

Our results confirm that EOR is the strongest independent predictor for OS in DLGG: patients who underwent radical resection (EOR = 100%), when functionally possible, showed significant survival benefit.

Association between postoperative tumor volume on T2-weighted MRI images and preoperative ΔT2T1 MRI index showed that the greater the preoperative ΔT2T1 MRI index, the less extensive was the possible resection; it highlights a possible predictive correlation between ΔT2T1 MRI index and EOR achievable as previously demonstrated^38^.

A major volumetric difference between T2-weighted and contrast-enhanced T1-weighted MRI sequences suggests a greater propensity of the tumor to have a diffuse growing pattern and consequently to be less resectable.

### The intraoperative surgical protocol

The principal limiting factor of surgical radicality lies in the infiltrating nature of these tumors, which gradually overgrow into eloquent areas both at cortical and subcortical level^14, 40^. A personalized anatomo-functional planning is consequently mandatory^6, 17^.

In the modern era of glioma surgery, increasing technological advances in imaging methods (such as intraoperative NN, MRI, and ultrasonography), stimulation mapping, and/or somatosensory-evoked potential techniques, are enabling surgeons to maximize cytoreduction and minimize morbidity.

Although there is growing literature supporting the accuracy and efficiency of individual techniques and methods^6, 11, 41^, we found no studies in literature that report the analysis of multimodal integrated protocols in the same cohort of DLGGs.

Volumetric studies have shown the prognostic significance of EOR for survival outcomes but have not compared the impact of the various intraoperative imaging modalities and surgical strategies in assisting/optimizing the maximal safe resection^13, 18^.

This investigation assessed the efficacy of the combination of different techniques in surgery of DLGGs that are positioned in or close to eloquent areas both in terms of morbidity and EOR.

Specifically, neuroradiological techniques, such as fMRI and 3D reconstruction of white matter tracts, represent useful tools to preoperatively analyze the three-dimensional relationships between the tumor and the neighboring cortical functional areas and subcortical pathways, respectively^6, 18, 42–44^. Intraoperative use of a guided navigation system enriched with functional and DTI data used in Series 2 significantly increase the possible EOR. Patients in Series 1 had a mean EOR of 83%, while those in Series 2 had a mean EOR of 88% (*p* < 0.001).

Overall, the use awake mapping and RTNT (*Protocol 3*) resulted in a higher EOR and lower permanent deficits. The data show that *Protocol 3* was superior to *Protocol 1* and *2* in improving the EOR. The median EOR and the number of radical resections is hence superior in patients belonging to *Protocol 3* in comparison with those belonging to *Protocol 1* and *2* (*p* < 0.001) (Fig. 1). Indeed, RTNT used in Protocol 3 proved to be an important step of the brain mapping technique.

Throughout surgical resection, the patient performs multiple complex tasks involving the cognitive function(s) related to the brain area where the surgeon is working at that moment, thus guiding the surgical team. When a significant decrement of performance is noted, the surgeon can stop resection or move to another area, otherwise the surgical resection can be carried out without the uncertainty of “classical” negative mapping. As a matter of fact, RTNT can be defined as a continuous neuropsychological monitoring.

Thus, a multimodal integrated protocol with those techniques (brain mapping and multimodal intraoperative imaging) and strategies (AS and RTNT) can improve the chances of an extensive resection, which has repercussions on survival and recurrence. In light of that, *Protocol 3* could represent a feasible support to the surgeon to safely maximize EOR in DLGGs surgery.

DES, both at cortical and subcortical levels, according to some experts of this field of surgery, represents the standard of care for DLGGs resection. It is used both for brain mapping and for monitoring neurologic performance, often in awake setting^13^.

Our study showed that patients who underwent AS had a better EOR in comparison to those operated under GA (*p* = 0.006) supporting the importance of AS for DLGGs in eloquent areas. This is the sole technique enabling a direct identification of neural networks and thus maximizing the resection while minimizing the risk of permanent deficits^39^. RTNT is based on a continuous neuropsychological testing administered during the surgical resection in order to fill the functional information gap between two consecutive DES, thus aiming at exploiting at its maximum the potentiality of AS^17^.

With regards to the neurological outcomes, our results showed that patients belonging to *Protocol 3* resulted in fewer late neurological deficits than those that underwent *Protocol 1*, even if the difference was not significant, which was mainly due to the limited sample size of this rare disease.

De Witt Hamer et al. hypothesized that AS enables more extended resection and improved tumor control, resulting in the preservation of neurological functions that can be mapped at the cost of early transient neurological deficits^13^. In this investigation, in line with previous studies, the simultaneous decrease in transient and permanent deficit testifies the preservation of functional subcortical pathways, ensuring the postoperative plasticity mechanism for the neurological recovery^45, 46^.

### The molecular landscape

The 2016 WHO classification incorporated molecular findings into brain tumor diagnosis. Phenotypic and genotypic parameters in the diagnosis of DLGGs (mutations in the *IDH1* and *IDH2* genes mutations and 1p*/*19q co-deletion) have become of crucial importance^47^.

This investigation confirms the role of molecular class as an independent predictor of OS^35, 48^. It also confirms the association between the molecular class and EOR achieved^22^.

The median EOR was larger in Oligodendroglioma than in Diffuse Astrocytoma IDH1/2 wild-type (92% vs. 83%; *p* = 0.002) and tended to also be larger in Diffuse Astrocytoma IDH1/2 mutated than in Diffuse Astrocytoma IDH1/2 wild-type (87% vs. 83%; *p* = 0.05). This difference could be related to a different tumor growing pattern.

Unfortunately, our data showed that DLGGs recurred in some of the patients even in cases of radical resection (Table 6). Interestingly, 3 and 5 years estimated OS in patients with an EOR of 100% was higher in patients belonging to molecular class of Diffuse Astrocytoma IDH1/2 mutated and Oligodendroglioma. Otherwise, considering the 10 years estimated OS, the percentage was similar amongst Diffuse Astrocytoma IDH1/2 mutated and Oligodendroglioma, and greater for Diffuse Astrocytoma IDH1/2 wild-type.

Future studies are thus required to better investigate the rich set of pertinent genomic and molecular markers, in addition to the radiological features in the era of radiogenomics, to better understand the biological behavior of DLGGs.

### Limitations

The retrospective nature of the investigation and the different treatments performed at tumor progression are the principal limitations of the present study. Nevertheless, we attempted to homogenize our data set by studying a large sample of patients based on strict inclusion criteria.

Furthermore, considering the low incidence of DLGGs, well-designed retrospective studies, even if limiting in nature, can contribute to the existing body of knowledge.

T2w-FLAIR sequences were not available for both pre- and postoperative measures for some patients. We used the late postoperative scan to minimize overestimation of postoperative tumor volume due to edema or ischemia and to minimize potential differences between sequences^49, 50^. Gliomas were classified according to the currently used WHO 2016 classification in this study, however, in light of the wide heterogeneity of DLGG, future investigations based on texture features from multi-parametric MRI and next generation sequences (NGS) analysis, may better clarify tumor recurrence even for those cases with a radical resection^47, 51^.

In addition, the cornerstone of LGGs surgery is based on the concept of maximal safe resection^5, 17, 39, 40^. Although intraoperative mapping of brain areas was shown to promote greater extent of resection and reduce functional deficits, this was shown only recently for some non-invasive techniques. We recognize that in this clinical setting the preoperative functional mapping by navigated transcraninical magnetic stimulation (nTMS) is an increasing valuable tool to stratify the individual risk of surgery-related deficits^52, 53^. Our presurgical work-up, at the moment, does not include this technique that has become an important tool in the modern surgical glioma management.

In closing, considering the higher interindividual variability of eloquent area arrangement and the wide potential displacement of functional sites, both at cortical level and subcortical pathways induced by the DLGG growing, a personal tailored multimodal integrated assessment of functional area should be developed to adopt in DLGG surgical field.

## Conclusions

Technological tools are clinically useful to improve the extent of resection. Multimodal integrated approaches combining awake craniotomy, RTNT, intraoperative brain mapping and functional imaging analysis can enhance maximal safe and efficient tumor resection with possible subsequent OS benefits.

Combining volumetric studies and Next Generation Sequence investigations will provide a better understanding of DLGG evolution, paving the way for molecular neurosurgery.

## References

[1] Capelle L (2013). Spontaneous and therapeutic prognostic factors in adult hemispheric World Health Organization Grade II gliomas: a series of 1097 cases: clinical article. J. Neurosurg..

[2] Clark VE, Cahill DP (2019). Extent of resection versus molecular classification: What matters when?. Neurosurg. Clin. N. Am..

[3] Claus EB (2005). Survival rates in patients with low-grade glioma after intraoperative magnetic resonance image guidance. Cancer.

[4] Hervey-Jumper SL, Berger MS (2016). Maximizing safe resection of low- and high-grade glioma. J. Neurooncol..

[5] Ius T (2017). An NF-κB signature predicts low-grade glioma prognosis: A precision medicine approach based on patient-derived stem cells. Neuro Oncol..

[6] Ius T (2012). Low-grade glioma surgery in eloquent areas: volumetric analysis of extent of resection and its impact on overall survival. A single-institution experience in 190 patients. J. Neurosurg..

[7] Lang FF, Gilbert MR (2006). Diffusely infiltrative low-grade gliomas in adults. J. Clin. Oncol..

[8] Nitta M (2015). Proposed therapeutic strategy for adult low-grade glioma based on aggressive tumor resection. Neurosurg. Focus.

[9] Sanai N (2019). How to build a neurosurgical oncology practice specializing in gliomas. Neurosurg. Clin. N. Am..

[10] Sanai N, Chang S, Berger MS (2011). Low-grade gliomas in adults. J. Neurosurg..

[11] Skrap M (2012). Surgery of insular nonenhancing gliomas: Volumetric analysis of tumoral resection, clinical outcome, and survival in a consecutive series of 66 cases. Neurosurgery.

[12] Smith JS (2008). Role of extent of resection in the long-term outcome of low-grade hemispheric gliomas. J. Clin. Oncol..

[13] De Witt Hamer PC, Robles SG, Zwinderman AH, Duffau H, Berger MS (2012). Impact of intraoperative stimulation brain mapping on glioma surgery outcome: A meta-analysis. J. Clin. Oncol..

[14] Mandonnet E (2014). Silent diffuse low-grade glioma: Toward screening and preventive treatment?. Cancer.

[15] Duffau H (2018). Is non-awake surgery for supratentorial adult low-grade glioma treatment still feasible?. Neurosurg. Rev..

[16] Duffau H (2018). Awake mapping is not an additional surgical technique but an alternative philosophy in the management of low-grade glioma patients. Neurosurg. Rev..

[17] Skrap M, Marin D, Ius T, Fabbro F, Tomasino B (2016). Brain mapping: a novel intraoperative neuropsychological approach. J. Neurosurg..

[18] Mazzucchi E, La Rocca G, Ius T, Sabatino G, Della Pepa GM (2020). Multimodality imaging techniques to assist surgery in low-grade gliomas. World Neurosurg..

[19] Berger MS, Ojemann GA (1992). Intraoperative brain mapping techniques in neuro-oncology. Stereotact. Funct. Neurosurg..

[20] Ius T, Angelini E, Thiebaut de Schotten M, Mandonnet E, Duffau H (2011). Evidence for potentials and limitations of brain plasticity using an atlas of functional resectability of WHO grade II gliomas: Towards a "minimal common brain". Neuroimage.

[21] Louis DN (2016). The 2016 World Health Organization classification of tumors of the central nervous system: A summary. Acta Neuropathol..

[22] Cesselli D (2019). Application of an artificial intelligence algorithm to prognostically stratify grade II gliomas. Cancers.

[23] Steyerberg E (2009). Clinical Prediction Models: A Practical Approach to Development, Validation, and Updating.

[24] Sanai N, Polley M-Y, Berger MS (2010). Insular glioma resection: Assessment of patient morbidity, survival, and tumor progression. J. Neurosurg..

[25] Majchrzak K (2012). The assessment of prognostic factors in surgical treatment of low-grade gliomas: A prospective study. Clin. Neurol. Neurosurg..

[26] Nitta M (2013). Updated therapeutic strategy for adult low-grade glioma stratified by resection and tumor subtype. Neurol. Med. Chir. (Tokyo).

[27] Snyder LA (2014). The impact of extent of resection on malignant transformation of pure oligodendrogliomas. J. Neurosurg..

[28] Coburger J (2016). Low-grade glioma surgery in intraoperative magnetic resonance imaging: Results of a multicenter retrospective assessment of the german study group for intraoperative magnetic resonance imaging. Neurosurgery.

[29] Jungk C (2016). Prognostic value of the extent of resection in supratentorial WHO grade II astrocytomas stratified for IDH1 mutation status: a single-center volumetric analysis. J. Neurooncol..

[30] Roelz R (2016). Residual tumor volume as best outcome predictor in low grade glioma—A nine-years near-randomized survey of surgery vs. biopsy. Sci. Rep..

[31] Eseonu CI (2017). Comparative volumetric analysis of the extent of resection of molecularly and histologically distinct low grade gliomas and its role on survival. J. Neurooncol..

[32] Eseonu CI, ReFaey K, Garcia O, Raghuraman G, Quinones-Hinojosa A (2017). Volumetric analysis of extent of resection, survival, and surgical outcomes for insular gliomas. World Neurosurg..

[33] Hameed NUF (2018). Transcortical insular glioma resection: clinical outcome and predictors. J. Neurosurg..

[34] Patel T (2018). The role of extent of resection in IDH1 wild-type or mutant low-grade gliomas. Neurosurgery.

[35] Wijnenga MMJ (2018). The impact of surgery in molecularly defined low-grade glioma: An integrated clinical, radiological, and molecular analysis. Neuro Oncol..

[36] Bo HK (2019). Intraoperative 3D ultrasound-guided resection of diffuse low-grade gliomas: Radiological and clinical results. J. Neurosurg..

[37] Morshed RA (2019). Molecular features and clinical outcomes in surgically treated low-grade diffuse gliomas in patients over the age of 60. J. Neurooncol..

[38] Ius T (2019). Incidental low-grade gliomas: Single-institution management based on clinical, surgical, and molecular data. Neurosurgery.

[39] Duffau H (2017). Diffuse Low-Grade Gliomas in Adults.

[40] Mandonnet E, Duffau H (2014). Understanding entangled cerebral networks: A prerequisite for restoring brain function with brain–computer interfaces. Front. Syst. Neurosci..

[41] Bello L (2010). Intraoperative use of diffusion tensor imaging fiber tractography and subcortical mapping for resection of gliomas: Technical considerations. Neurosurg. Focus.

[42] Raffa G (2018). The impact of diffusion tensor imaging fiber tracking of the corticospinal tract based on navigated transcranial magnetic stimulation on surgery of motor-eloquent brain lesions. Neurosurgery.

[43] Campanella M, Ius T, Skrap M, Fadiga L (2014). Alterations in fiber pathways reveal brain tumor typology: A diffusion tractography study. PeerJ.

[44] de Bertoldi F (2015). Improving the reliability of single-subject fMRI by weighting intra-run variability. Neuroimage.

[45] Duffau H, Taillandier L (2015). New concepts in the management of diffuse low-grade glioma: Proposal of a multistage and individualized therapeutic approach. Neuro Oncol..

[46] Rossi M (2019). Is supratotal resection achievable in low-grade gliomas? Feasibility, putative factors, safety, and functional outcome. J. Neurosurg..

[47] Louis DN (2019). cIMPACT-NOW: A practical summary of diagnostic points from Round 1 updates. Brain Pathol.

[48] Brat DJ (2018). cIMPACT-NOW update 3: Recommended diagnostic criteria for "Diffuse astrocytic glioma, IDH-wildtype, with molecular features of glioblastoma, WHO grade IV". Acta Neuropathol..

[49] Ius T (2014). Surgery for insular low-grade glioma: Predictors of postoperative seizure outcome. J. Neurosurg..

[50] Pala A (2016). The value of intraoperative and early postoperative magnetic resonance imaging in low-grade glioma surgery: A retrospective study. World Neurosurg..

[51] Yang Y (2019). Optimizing texture retrieving model for multimodal MR image-based support vector machine for classifying glioma. J. Magn. Reson. Imaging (JMRI).

[52] Raffa G (2019). The role of navigated transcranial magnetic stimulation for surgery of motor-eloquent brain tumors: A systematic review and meta-analysis. Clin. Neurol. Neurosurg..

[53] Ille S, Krieg SM (2021). Functional mapping for **glioma** surgery, part 1: Preoperative mapping tools. Neurosurg. Clin. N. Am..

